# Effectiveness of the school-based oral health promotion programmes from preschool to high school: A systematic review

**DOI:** 10.1371/journal.pone.0256007

**Published:** 2021-08-11

**Authors:** Taufan Bramantoro, Cornelia Melinda Adi Santoso, Ninuk Hariyani, Dini Setyowati, Amalia Ayu Zulfiana, Nor Azlida Mohd Nor, Attila Nagy, Dyah Nawang Palupi Pratamawari, Wahyuning Ratih Irmalia

**Affiliations:** 1 Department of Dental Public Health, Faculty of Dental Medicine, Universitas Airlangga, Surabaya, Indonesia; 2 Dental and Oral Health Committee, Ministry of Health Republic of Indonesia, Jakarta, Indonesia; 3 Faculty of Public Health, University of Debrecen, Debrecen, Hungary; 4 Department of Community Oral Health and Clinical Prevention, Faculty of Dentistry, University of Malaya, Kuala Lumpur, Malaysia; 5 Postgraduate Program, Faculty of Dental Medicine, Universitas Airlangga, Surabaya, Indonesia; 6 Indonesian Health Innovation and Collaboration Institute, Surabaya, Indonesia; Forsyth Institute, UNITED STATES

## Abstract

**Background:**

Schools offer an opportunity for oral health promotion in children and adolescents. The purpose of this study was to conduct a systematic review of the influence of school-based oral health promotion programmes on oral health knowledge (OHK), behaviours (OHB), attitude (OHA), status (OHS), and quality of life (OHRQoL) of children and adolescents.

**Methods:**

A systematic search on the PubMed and Embase databases was conducted to identify eligible studies. The last search was done on April 24^th^, 2020. The quality of the included studies was evaluated using the Joanna Briggs Institute (JBI) Critical Appraisal tools.

**Results:**

Of the 997 articles identified, 31 articles were included in this review. Seven studies targeted students in preschools, seventeen in elementary schools, and seven in high schools. Most of these studies revealed positive outcomes. Some studies showed that the school-based oral health promotion programmes showed better OHK, OHB, OHS, and OHRQoL.

**Conclusion:**

Positive results were obtained through oral health promotion programmes in schools, especially those involving children, teachers, and parents.

## Introduction

Oral diseases pose a significant public health challenge, especially among children and adolescents. Around 60–90% of school children worldwide suffered from caries [1] and over 531 million children had caries of deciduous teeth [2]. Moreover, most children and adolescents showed gingivitis symptoms. Approximately 2% of youth had aggressive periodontitis, which might lead to premature tooth loss [1]. Oral diseases can negatively affect the quality of life, cause pain, limitation in oral functions, impaired nutrition, emotional stress, low self-esteem, and poor school attendance and performance [3–6]. They also impose a considerable economic burden as oral health treatments are often expensive. The treatment cost of dental caries alone for children was estimated to surpass the total budget of healthcare for children in low-income countries [7].

One of the efforts to improve the oral health of children and adolescents is by implementing school-based oral health promotion programmes, as proposed by the World Health Organisation (WHO) [8]. Schools serve as ideal settings for health promotion as they can reach most school-aged children and provide important networks to their families and communities [8, 9]. School-based programs can also help increase children’s access to dental services, especially those from disadvantaged socio-economic backgrounds [10]. Moreover, school years cover the life period of childhood and adolescence, during which lifelong sustainable behaviours, beliefs, and attitudes related to health are established [8].

Several school-based oral health promotion programmes have been proposed, such as oral health education (OHE), tooth-brushing activities, the provision of fissure sealant, or other treatments [11, 12]. While the effectiveness of the programs has been investigated, extensive evidence from a global viewpoint is still limited. Moreover, existing systematic reviews only focused on OHE [13–15]. A study providing a complete picture of the effectiveness of different kinds of oral health programmes at various school settings has not yet been available. This information is necessary to help the development of policies and the allocation of resources [13].

The objective of this study was to systematically review the effectiveness of the school-based oral health promotion programmes on oral health knowledge (OHK), behaviours (OHB), attitude (OHA), status (OHS), and quality of life (OHRQoL) of children and adolescents at preschools, elementary schools, and high schools.

## Materials and methods

We systematically reviewed a series of published articles to answer the question–What is the significance of school-based oral health programmes on children and adolescents?

We chose the eligible articles according to the following criteria:

1. All types of experimental studies (randomised controlled trials, quasi-experimental studies)
2. Written in English;
3. Study subjects were pre-schoolers, school children, and school adolescents;
4. The intervention included all types of oral health intervention programmes conducted in preschools, elementary schools, or high schools;
5. The outcome was OHK, OHB, OHA, OHS, and OHRQoL.

There was no limitation on publication year. Protocols, reviews, editorial letters, and commentaries were excluded.

### Search strategy

PubMed and Embase were chosen as the database sources for our study, as they are considered to be the largest pharmaceutical and biomedical databases. The last search was on April 24^th^, 2020. We used search terms related to oral health promotion, school, children, adolescents, randomised controlled trial, quasi-experimental study, OHK, OHB, OHA, OHRQoL, oral hygiene, and oral diseases, such as caries, periodontitis, and toothache.

### Study selection, data extraction, quality assessment

Two independent reviewers performed the study selection, data extraction, and assessment of the quality of studies. After the records were obtained from the databases and duplicates were eliminated, the titles and abstracts were screened based on the selection criteria. A full-text review was then conducted to identify eligible studies. Data of the included studies was recorded (i.e., author, publication year, country, school setting, study population, interventions, comparator or control group, and results). The quality of the included studies was evaluated using the Joanna Briggs Institute (JBI) Critical Appraisal tools for randomised controlled trials and quasi-experimental studies [16]. Any disagreements or ambiguities were resolved through discussion.

## Results

**Fig 1:**
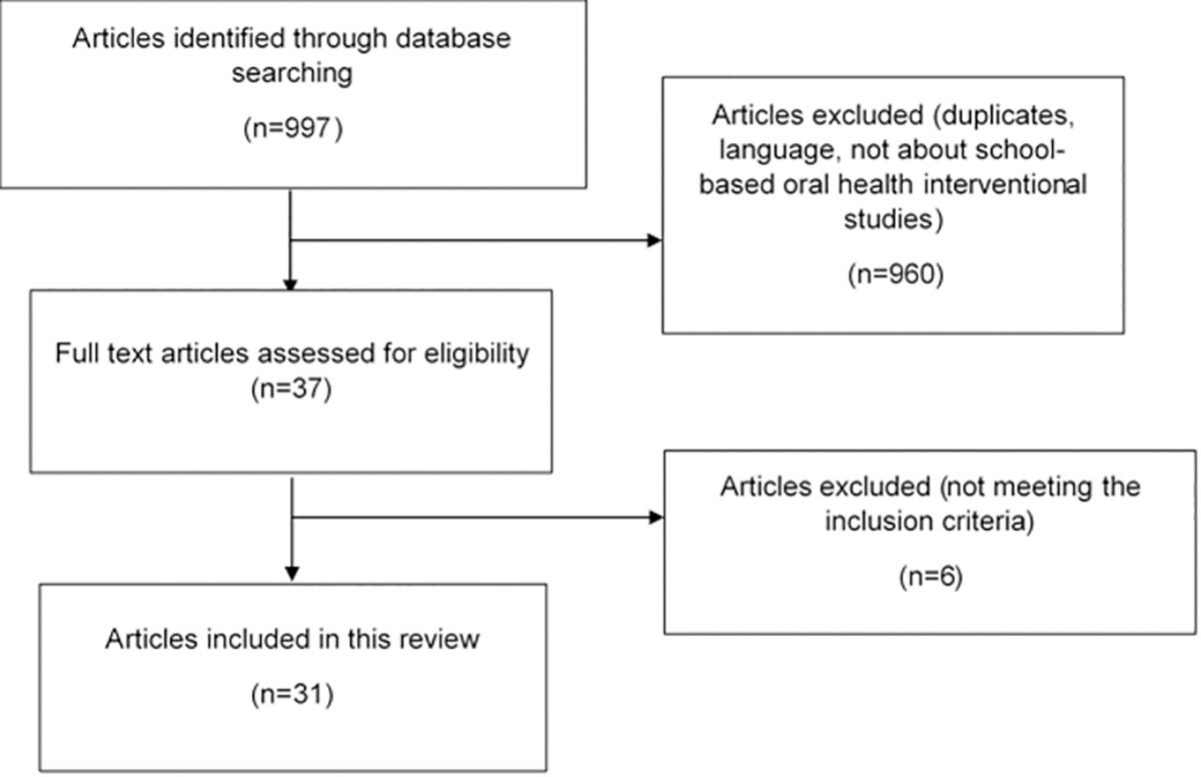
The flow diagram of the study selection process.

A total of 997 records were obtained from the databases. After removing duplicates and screening titles and abstracts, 37 articles remained for the full-text review. Of these, 31 studies met the eligibility criteria and were included in our review. The flow diagram of the study selection process can be seen in Fig 1.

### Characteristics of the studies

The included studies in this review were from four distinct regions, which were Asia, Europe, Africa, and America. The two largest proportions were from Asia (48%) and Europe (26%). Of the 31 studies included, four were from the United Kingdom; 3 of each were from the following countries: Iran, Brazil, China; 2 of each were from the following countries: India, Pakistan, Hong Kong, and Germany; and one of each was from the following countries: Myanmar, Thailand, Turkey, Switzerland, Sweden, Argentina, the United States, Nigeria, Tanzania, and Zimbabwe. The publication year varied from 1976 to 2019. Twenty-seven studies used randomised clinical trial designs, while four studies used quasi-experimental designs. Seven studies targeted the student populations in preschools, seventeen studies in elementary schools, and seven studies in high schools. All the included studies had sufficient methodological quality.

#### The effects of school-based oral health promotion programmes on children

*1*. *Preschool children*. Table 1 shows the summary of studies conducted in preschools. Intervention in all studies involved delivering oral health information to children. OHE for teachers was conducted in three studies [17–19], and for parents in two studies [18, 19]. One study investigated the effectiveness of education through games and puppet shows [20], one study on the methods of education (either delivered by a teacher, a dentist, or role-playing dental residents) [21], one study on a specific tooth-brushing instruction [22], and one study on professional cross-brushing on first permanent molar surfaces [23]. Four studies included supervised tooth-brushing [17–19, 23], two studies included the provision of fluoridated toothpaste and toothbrushes [17, 18], and one study included the application of sodium fluoride phosphate [19] as part of their interventions.

**Table 1:** The summary of studies conducted in preschools.

No	Author, country, year	Intervention type	Study population	Aims	Outcome measures	Important results
1	Makuch and Reschke, Germany, 2001 [20]	The use of a series of games and exercises to convey dental health information; compared to verbal instructions.	3–6 years old children.	To find a new way for dental health education, which is via games.	Knowledge and tooth-brushing skills.	The use of games and shows aimed at the developmental level of the children was more effective than verbal instructions in improving oral hygiene knowledge and skills.
2	You et al., China, 2002 [17]	The use of 1100 ppm sodium fluoride dentifrice, supervised toothbrushing, OHE for children and teachers; compared to the provision of placebo dentifrice and no program.	3 years old children.	To examine the effects of an 1100 ppm sodium fluoride dentifrice in the context of a kindergarten-based oral health program.	dmfs increment score.	Fluoride in conjunction with increased dental awareness can deliver important reductions in caries.
3	Rong et al., China, 2003 [18]	OHE to children, teachers, and parents, supervised toothbrushing, provision of fluoridated toothpastes and toothbrushes; compared to the provision of non-fluoridated toothpastes, toothbrushes, and no program.	3 years old children.	To evaluate a 2-year oral health education and caries prevention program in kindergartens.	dmfs and oral health habits of the children, OHK and OHA of their parents.	The program was effective in reducing the development of new dental caries, establishing good oral health habits of the children, and increasing OHK and OHA of their parents.
4	Hochstetter et al., Argentina, 2007 [19]	The provision of educational (OHE for children, teachers, and parents) and preventive programs (application of sodium fluoride phosphate, supervised toothbrushing with fluoride); compared to the provision of preventive program only.	3.5–5 years old children.	To evaluate the impact of the preventive educational programme in pre-schoolers.	dmfs, dmft, gingival index, and plaque index.	The inclusion of an educational component significantly increases the effectiveness of measures aimed at preventing caries and gingivitis.
5	Ramseier et al., Switzerland, 2007 [22]	A 15-minutes health education programme on the importance of body cleanliness for all subjects, followed by additional oral hygiene instruction for half of the subjects, while hand and fingernail hygiene instructions for the other half.	5–7 years old children.	To compare the result between a short (15 minutes) oral hygiene education and hand hygiene education.	Plaque control record, nail hygiene index, and hand hygiene index.	The provision of oral hygiene instruction significantly improved the children’s oral hygiene.
6	Frazão, Brazil, 2011 [23]	The provision of conventional program and professional cross-brushing on surfaces of first permanent molar rendered by a trained dental assistant five times per year; compared to the provision of conventional program only.	5 years old children.	To assess if the bucco-lingual technique can increase the effectiveness of a school-based supervised toothbrushing program on preventing caries.	dmft.	The modified program was effective in reducing caries incidence among the boys.
7	John et al., India, 2013 [21]	Group A (OHE from the dentist); Group B (OHE from the class teacher trained by the dentist); Group C (OHE from the dental residents dressed to imitate cartoon characters, accompanied with audio-visual effects); compared to group D (without any health education interventions).	4–6 years old children.	To assess the impact of three different health education methods among pre-schoolers.	Debris index.	Delivering OHE via drama made a better oral hygiene improvement than conventional educations.

Note: OHE = oral health education; OHA = oral health attitude; OHK = oral health knowledge; dmft = decayed, missing, filled deciduous teeth; dmfs = decayed, missing, filled deciduous teeth surfaces.

Delivering education through games and shows resulted in significantly better oral hygiene knowledge and skills than verbal instructions [20]. Children receiving a role-playing or drama mode of health education had significantly better oral hygiene than those without interventions or those receiving conventional education from a dentist or a trained teacher [21]. A specific instruction on oral hygiene is proven to significantly improve children’s oral hygiene [22]. The addition of educational programmes for parents, teachers, and children as a support to the preventive programmes (application of sodium fluoride phosphate, supervised toothbrushing with fluoride) led to the significant reductions in gingival index and plaque index scores and no changes in dmft and dmfs scores. Meanwhile, the group without the addition of educational programmes showed significant increases in gingival index, plaque index, dmft, and dmfs scores [19].

Compared to the control group, the group which received a school programme covering OHE for children, teachers, and parents, a supervised toothbrushing, and provision of fluoridated toothpaste and toothbrushes had 30.6% lower dmfs increment and a higher percentage of children brushing twice a day [18]. A similar programme, comprising of OHE for children and teachers, supervised tooth brushing, and the use of 1100 ppm fluoride dentifrice, also led to a significantly lower dmfs increment than the control group [17]. Among boys, the school-based supervised tooth-brushing programme that also covered professional cross-brushing on the first permanent molar surfaces led to 50% lower caries incidence density compared to the group receiving only the conventional tooth-brushing programme at school [23].

*2*. *Elementary school children*. Table 2 shows the summary of studies conducted in elementary schools. Six studies focused on the effectiveness of the OHE programmes [11, 24–28], one study on the importance of repetition and reinforcement [29], three studies on supervised toothbrushing [30–32], one study on tooth-brushing training [33], one study on school dental screening [34], and two studies on SOC-based interventions [35, 36]. Besides involving education as part of the interventions, one study further included dietary counselling, the ingestion of fluoridated drinking water, and supervised toothbrushing [37], one study included a dental hospital tour programme [12], two studies included the provision of preventive and restorative care [12, 37], three studies included the provision of oral hygiene aids [12, 25, 37], and two studies included competition activities [12, 38].

**Table 2:** The summary of studies conducted in elementary schools.

No	Author, country, year	Intervention type	Study population	Aims	Outcome measures	Important results
1	Bagramian et al., the United States, 1976 [37]	The provision of 5 preventive and therapeutic measures (fluoridated drinking water, OHE including supervised toothbrushing, dietary counselling, dental examinations, application of sealant to posterior teeth, and the provision of all necessary restorative care), compared to the provision of only 3 measures (fluoridated drinking water, OHE, including supervised toothbrushing, dietary counselling, and dental examinations).	6–17 years old children.	To determine the caries-preventive benefit provided by a combination of 5 preventive and therapeutic measures.	Caries increment.	The comparison group had significantly higher caries increment than the intervention group.
2	van Palenstein Helderman et al., Tanzania, 1992 [30]	A program consisting of OHE, brushing session, regular visit by a dental team member, and the provision of curative dental care.	10–13 years old children.	To evaluate oral hygiene of habitual chewing stick and toothbrush users who participated in an OHE programme in schools.	Plaque and gingival bleeding scores.	The program significantly improved oral hygiene, regardless of the oral hygiene tools used.
3	Zarod and Lennon, the United Kingdom, 1992 [34]	A school dental screening, combined with a thorough referral and follow-up (sending a letter to parents via their child, by mail or phone); compared to no communication after screening.	4–6 years old children.	To determine the effectiveness of a school dental screening in encouraging school children aged 4 to 6 years to visit a dentist.	Dental attendance.	Following screening, a series of follow-up communication to encourage parents taking their children to a dentist was effective in increasing dental attendance of school children.
4	Albandar et al., Brazil, 1994 [25]	Group 1 (comprehensive needs-related oral hygiene training program, which was based on individual needs, including OHE for parents and teachers, and the provision of toothbrushes and fluoridated toothpastes); Group 2 (conventional oral hygiene training program, which was less comprehensive and without parental participation, but with the provision of toothbrushes and fluoridated toothpastes); Group 3 (no program, the provision of fluoridated toothpastes only).	13 years old children.	To evaluate the efficacy of self-performed preventive programs on the control of plaque and the prevention of gingival inflammation in adolescents.	Plaque index, the presence of gingival bleeding.	The comprehensive group showed significantly better improvement in oral hygiene and gingival health than the control group. Results from the less comprehensive group were not significantly different from the control group.
5	Frencken et al., Zimbawe, 2001 [26]	Schools with teachers attending a 3-day workshop about oral health and rehabilitation.	8–10 years old children.	To assess the effectiveness of an oral health education programme administered by schoolteachers in a district in Zimbabwe over a period of 3.5 years.	Plaque accumulation and caries increment.	One-time training of teachers was ineffective in reducing plaque levels. Its effect on caries levels was inconclusive, considering the low caries increment observed over the study period.
6	Jackson et al., the United Kingdom, 2005 [31]	Daily teacher-supervised toothbrushing at school with fluoridated toothpastes.	5–6 years old children.	To determine whether teacher-supervised toothbrushing, once a day, at school, during term time, with commercial toothpaste containing 1450 ppm fluoride, could reduce dental caries in primary school children when compared with children from the same community who did not receive this intervention.	Caries increment	The overall caries increment of children in the intervention group was significantly less than those in the non-intervention group.
7	Saied-Moallemi et al., Iran, 2009 [28]	Group 1 (intervention via class work); Group 2 (intervention via parents); Group 3 (intervention via class work and parents); compared to a group without intervention.	9 years old children.	To evaluate the effectiveness of a school-based oral health promotion intervention on preadolescents’ gingival health.	Dental plaque and gingival bleeding.	Parental-aid and combined groups had better oral hygiene and gingival health status than the control group. Outcomes in the class-work group did not differ from those in the control group.
8	Tai et al., China, 2009 [12]	A 3-year program, consisting of a 30-minute OHE for children delivered by teachers biweekly, a 30-minute OHE for mothers annually, OHE booklet for children, annual presentation of OHE posters, contests on OHK, a tour of the dental hospital, oral examination by dentists in the classrooms annually, provision of fluoride toothpaste once every 2 months, and provision of preventive and curative care; compared to no program.	6–7 years old children.	To assess the outcome of oral health promotion in school children over a 3-year period in Yichang City, Hubei, China.	Caries increment (DMFT, DMFS), oral hygiene status, oral care habits, and the variable “restoration, sealant, and decay”.	The intervention group had a lower mean DMFS increment score, higher reductions in plaque and sulcus bleeding scores, higher scores in restorations and sealants received, a lower score in untreated caries, and more favourable OHB, than the control group. There was no significant difference in mean DMFT increment score between the groups.
9	Yekaninejad et al., Iran, 2012 [11]	The comprehensive group (intervention to encourage children, parents, and school staffs to increase the frequency of toothbrushing and flossing); the student group (intervention targeted only children); compared to the control group (no intervention).	11–12 years old children.	To investigate whether an intervention targeting parents and school staffs can improve OHB and OHS of school children.	OHB (brushing and flossing), oral hygiene, Community Periodontal indices, and Health Belief Model components.	Students in the comprehensive intervention group had better OHB, oral hygiene, and gingival health status, than those in the student intervention or control groups.
10	Çalişir et al., Turkey, 2012 [33]	A training program on tooth-brushing skills, comprising of seven basic steps of teaching skills; compared to no program.	9–10 years old children.	To evaluate the effects of individual training on tooth brushing skills of primary school children.	Brushing skills.	Children in the intervention group had significantly higher post-training test scores than those in the control group.
11	Rosema et al., Myanmar, 2012 [32]	A daily school-based toothbrushing programme; compared to no programme.	8–11 years old children.	To assess whether gingivitis and plaque scores of 8- to 11-year-old school children who participated in the programme for 2 years were lower than those who did not participate in the programme.	Bleeding on marginal probing index, Quigley & Hein plaque index.	The programme did not have significant effects on gingivitis and plaque scores.
12	Haleem et al., Pakistan, 2012 [27]	Dentist-led OHE group; Teacher-led OHE group; Peer-led OHE group; Self-learning group; compared to a control group without any form of OHE.	10–11 years old children.	To compare the effectiveness of dentist-led, teacher-led, peer-led, and self-learning strategies of OHE.	Oral hygiene status (plaque, bleeding on probing, calculus), OHK and OHB about gingivitis and oral cancer.	The dentist-led, teacher-led, and peer-led OHE were equally effective in improving OHK and oral hygiene status. The peer-led OHE was almost as effective as the dentist-led OHE and comparatively more effective than the teacher-led and self-learning strategies in improving OHB.
13	Nammontri et al., Thailand, 2012 [36]	SOC intervention delivered by trained teachers; compared to no intervention.	10–12 years old children.	To test the effects of an intervention to enhance SOC on OHRQoL in children.	SOC, OHRQoL, oral health beliefs, gingival health score.	The intervention improved SOC, OHRQoL, oral health beliefs, and gingival health.
14	Freeman et al., the United Kingdom and Ireland, 2015 [38]	The Winning Smiles school-based toothbrushing programme, consisting of an oral health promoter component, a teacher component, and an award ceremony.	7–8 years old children.	To use a model of health learning to examine the role of health-learning capacity and the effect of a school-based oral health education intervention (Winning Smiles) on the health outcome, child OHRQoL.	Child OHRQoL, self-esteem, knowledge on toothbrushing and fluoride toothpaste, and salivary fluoride level.	The intervention had a significant effect on toothbrushing–fluoride toothpaste knowledge and a borderline effect on child OHRQoL. Knowledge was strongly associated with saliva fluoride concentration.
15	Haleem et al., Pakistan, 2016 [29]	The dentist-led, teacher-led, and peer-led groups received a single OHE session and were evaluated post-intervention and 6 months after. The three groups were then exposed to OHE for 6 months, followed by 1 year of no OHE activity.	10–11 years old children.	To determine the effectiveness of the repeated and reinforced OHE compared to one-time OHE and to assess its role in school-based OHE imparted by dentist, teachers and peers.	OHK, OHA, OHB, DMFT, and oral hygiene status (plaque, bleeding on probing, calculus).	The repeated and reinforced OHE significantly increased OHK, OHB, and oral hygiene status indices at 6-month evaluation of reinforcement phase, irrespective of the OHE strategy. Although the OHK scores of the dentist-led and peer-led groups decreased significantly at 12-month evaluation of reinforcement phase, the said score of the teacher-led group; and OHB and oral hygiene status scores of all three groups remained statistically unchanged during this period.
16	Qadri et al., Germany, 2018 [24]	Oral health promotion was integrated into a general health promotion program and school curricula and activities, delivered by teachers.	9–12 years old children.	To evaluate the effects of 1.5 years of an oral health promotion program in primary schools.	DMFT, caries increment, OHK, OHA, and OHB.	The program was effective in reducing caries incidence in high SES groups, whereas no preventive effect was found in low SES groups. OHK, OHA, and OHB did not change appreciably during the study period.
17	Tomazoni et al., Brazil, 2019 [35]	A 2-month SOC intervention delivered by trained teachers; compared to no intervention.	8–14 years old children.	To test the effectiveness of a school-based intervention to enhance the SOC and OHRQoL of socially vulnerable Brazilian children.	OHRQoL and SOC.	The intervention was effective in improving SOC and OHRQoL.

Note: OHE = oral health education; OHK = oral health knowledge; OHB = oral health behavior; OHS = oral health status; OHRQoL = oral health-related quality of life; DMFT = decayed, missing, filled permanent teeth; DMFS = decayed, missing, filled permanent teeth surfaces; SOC = sense of coherence; SES = socioeconomic status.

OHE that was incorporated into a school curriculum lowered the risk of developing new carious lesions by 35%. However, the effect was modified by parental socioeconomic status (SES) since high SES in the intervention group was associated with a 94% incidence rate ratio (IRR) reduction [24]. One-time teacher training on oral health did not significantly make differences in means of plaque and caries increment scores compared to the control group [26].

A programme consisting of OHE, teacher supports, and competition had a significant effect on OHK and an effect on OHRQoL [38]. Those with a comprehensive programme of OHE for children and parents, a contest, dental hospital tour, oral examination, provision of fluoride toothpaste, and preventive and curative treatments showed significantly lower DMFS increment mean score, untreated dental caries scores, higher reductions in plaque and sulcus bleeding scores, higher proportions in restoration and sealants, and showed changes towards good practices of oral care compared to the control group [12]. Children receiving a comprehensive needs-related oral hygiene training programme had significantly less gingival bleeding and plaque than the control group, whereas there were no differences found between the less comprehensive group and the control group [25]. Children with a comprehensive OHE targeted for them, their parents, and teachers had significantly better OHB, oral hygiene, and gingival health status than other groups. Children with OHE targeted for only them had significantly better OHB and oral hygiene than the control group, but there was no difference in terms of gingival health [11]. OHE via parents at home or the combination between parental involvement and class activities significantly improved oral hygiene and gingival health status compared to the control group. Meanwhile, no significant differences were observed between the class-work group and the control group [28].

Groups receiving OHE led by dentists, teachers, or peers had significantly better OHK, OHB, and oral hygiene status than self-learning or control groups. There were no significant differences in OHK and oral hygiene status between the three educator-led groups. Nevertheless, the peer-led group had a significantly better OHB than the teacher-led group. The self-learning group had a significantly better OHB than the control group, but there were no differences in OHK and oral hygiene status between them [27].

One-time OHE session had no significant effect on oral hygiene status, regardless of the educators. One-time dentist-led and peer-led OHE sessions significantly increased OHK and OHB related to gingivitis, but there was no significant change in OHB related to oral cancer. One-time teacher-led OHE session had no significant effects on OHK and OHB. However, six months after repeated and reinforced OHE (RR-OHE), the OHK, OHB, and oral hygiene status significantly improved, regardless of the educators. Although 12 months after the RR-OHE, the OHK of the dentist-led and peer-led groups significantly decreased, there were no significant changes in the OHK of the teacher-led group, as well as in the OHB and oral hygiene status of all the groups [29].

An individual tooth-brushing training programme significantly improved children’s brushing skills compared to the control group [33]. Children receiving a programme of tooth brushing with fluoride toothpaste supervised by teachers had a significantly less overall caries increment than those in the control group [31]. The provision of brushing sessions from trained teachers and curative dental care on-demand significantly reduced the plaque and gingival bleeding scores. The reductions of scores were comparable between chewing stick and toothbrush users [30]. One quasi-experimental study in Burma found that a school-based tooth-brushing programme had no significant effects on plaque and bleeding scores [32].

Children receiving a 2-month sense of coherence (SOC) intervention from trained teachers had significantly better OHRQoL and SOC improvement than the control group [35]. Another study also found that the SOC intervention group had significantly better OHRQoL, SOC, oral health beliefs, and gingival health than the control group [36]. The provision of five preventive and therapeutic measures significantly reduced caries increment compared to the provision of three preventive measures only [37]. School dental screening, followed by a series of communication to encourage parents into taking their children to a dentist significantly improved dental attendance [34].

*3*. *High school children*. Table 3 shows the summary of studies conducted in high schools. Two studies investigated the effectiveness of education through posters or pamphlets [39, 40]. Besides including education as part of the interventions, one study further explored the effectiveness of the provision of oral hygiene aids [41] and one study on the use of the different types of oral hygiene instruments [42]. There was one quasi-experimental study on the evaluation of the Natural Nashers programme in England [43], one study on the effectiveness of motivational interviewing [44], and one study on the involvement of dental hygienists at schools (education, open clinic, including fluoride varnish treatments) [45].

**Table 3:** The summary of studies conducted in high schools.

No	Author, country, year	Intervention type	Study population	Aims	Outcome measures	Important results
1	Craft et al., the United Kingdom, 1984 [43].	Natural Nashers program (a 3-week program designed to be integrated into the third-year Biology curriculum using three 70–80-minute sessions, containing a key lesson (slide presentation of information), a class experiment (activity and participation), and pupil worksheets (reinforcement), the provision of personal dental health kits and special diaries of activities (recording personal plaque removal, monitoring the diet, interviewing family members, counting the teeth of siblings)).	13–14 years old children.	To motivate adolescents to carry out effective and efficient oral hygiene and to choose safe snacks between meals, as part of an integrated curriculum experience.	OHK, OHA, plaque and gingival scores.	The program improved OHK and OHA, and reduced plaque and gingival scores.
2	Sote, Nigeria, 1991 [42].	A 2-week oral health education programmes, followed by the provision of toothbrushes and fluoridated toothpastes for group A, chewing stick Sorendeia warneckei for group B, and chewing stick Massularia acuminata for group C.	12–14 years old children.	To educate children on good oral health maintenance and the use of various types of oral hygiene, and to evaluate the impact of this knowledge on gingival health.	Plaque scores.	More toothbrush users than chewing stick users had gingivitis.
3	Young et al., Hong Kong, 2014 [39].	A 2-week display of posters of dental trauma management; compared to no display of such posters.	11–19 years old children.	To investigate the effectiveness of educational poster on improving secondary school students’ knowledge of emergency management of dental trauma.	Knowledge of dental trauma.	Educational poster on dental trauma management significantly improved students’ knowledge.
4	Chandrashekar et al., India, 2014 [41].	Group 1 (no OHE after the initial health education at the time of screening); Group 2 (OHE by a dentist at 3 months interval using the audio-visual aids); Group 3 (OHE by trained schoolteachers with screening for gross calculus deposits, debris, etc. on a fortnightly basis); Group 4 (the same treatment as group 3, but with the addition of the provision of toothbrushes and toothpastes).	15 years old children.	To compare oral hygiene, plaque, gingival, and dental caries status of rural children receiving OHE by dentists and schoolteachers with and without supply of oral hygiene aids.	OHI-S, PI, GI, and DMF-S.	Frequent OHE combined with the provision of oral hygiene aids made the highest reduction in OHI-S, PI, and GI scores.
5	Pakpour et al., Iran, 2013 [40].	The gain- and loss-framed pamphlets each contained six positive or negative messages and three related full-colour images, which were allowed to be taken home at the end of session (no discussion took place).	15 years old children.	To examine the effects of two message framing interventions on oral self-care behaviours and health among Iranian adolescents.	Brushing/flossing behaviour, cognitive (attitudes, intentions), OHRQoL, dental plaque, and periodontal status.	Loss-framed messages were more effective than gain-framed messages in encouraging oral self-care behaviours. These effects were mediated through attitudes and intentions.
6	Hedman et al., Sweden, 2015 [45].	Health education and preventive measures, such as fluoride varnish treatments every 6 months (carried out by dental hygienists that worked 4 hours every week at schools for two years); compared to no intervention.	12–16 years old children.	To investigate the possibility of influencing adolescents’ caries incidence, knowledge and attitudes towards oral health and tobacco through a school-based oral health intervention programme.	Caries incidence, knowledge and attitudes towards oral health and tobacco use.	The intervention had limited impacts on caries incidence, knowledge, and attitudes, but it seemed to increase adolescents’ interests in oral health.
7	Wu et al., Hong Kong, 2017 [44].	Group 1 (prevailing health education); Group 2 (motivational interviewing); Group 3 (motivational interviewing coupled with interactive dental caries risk assessment).	12–13 years old children.	To evaluate the effectiveness of motivational interviewing in improving adolescents’ oral health.	Oral health self-efficacy, behaviours, plaque score, and dental caries status.	Motivational interviewing was more effective than prevailing health education strategy in improving OHB and preventing caries.

Note: OHE = oral health education; OHA = oral health attitude; OHB = oral health behaviours; OHK = oral health knowledge; OHRQoL = oral health-related quality of life; OHI-S = simplified oral hygiene index; PI = plaque index; GI = gingival index; DMFS = decayed, missing, filled permanent teeth surfaces.

A two-week display of educational posters concerning dental trauma significantly improved knowledge on dental trauma management [39]. Children receiving a loss-framed pamphlet intervention had better OHB, attitude, and intention to brush at a 2-week follow-up, less dental plaque, better OHRQOL, and gingival health at a 24-week follow-up compared to other groups [40]. The Natural Nashers programme generally reduced children’s plaque and gingival scores and improved their OHK and OHA compared to the control group [43]. Frequent teacher-led OHE sessions along with the provision of oral hygiene aids significantly reduced simplified oral hygiene index (OHI-S), plaque index (PI), and gingival index (GI) scores. In contrast, these scores significantly increased among those receiving infrequent dentist-led OHE sessions or those without intervention. There was no pre-post difference in mean DMF-S score for all groups [41].

Dental hygienists working in schools to deliver OHE and preventive measures (fluoride varnish treatments) impacted the incidence of enamel caries, but there was no effect on dentin caries. The intervention also improved OHK and oral hygiene, but there was no effect on attitudes toward tobacco [45]. Following OHE programme, children who were assigned to use toothbrushes had a higher gingivitis occurrence than those assigned to use chewing sticks in Nigeria [42]. Children receiving a motivational interviewing session had a lower number of new carious teeth, tended to reduce snacking, and increased their tooth-brushing frequency compared to those who received a traditional OHE. The inclusion of caries risk assessment into motivational interviewing provided additional effects only on oral hygiene, but not on the other outcomes [44].

## Discussion

This study was among the few to provide a comprehensive summary of the effectiveness of oral health promotion programmes in different school settings, ranging from preschools to high schools. One of the limitations was the restriction to take into account only the studies published in English, which might cause language bias. The search for conference proceedings, dissertations, and unpublished studies was not performed. It was challenging to summarise the findings of the studies due to high variabilities in the type and method of interventions, outcome measurements, and age of the samples. Thus, it was not feasible to provide a quantitative comparison, as reported by a previous review [15]. The strategy or design of oral health promotion programs rather varies across countries, depending on the financing and planning of the health and education sectors, the socioeconomic condition, culture, and the burden of oral diseases in the country [46].

According to WHO, schools are ideal settings to promote oral health. An individual spends most of their childhood and adolescence time at schools. This period is a critical stage of the life course, during which behavioural patterns are built, and that may indicate their future health status. Moreover, children can learn new information rapidly at this stage. The sooner habits are formed, the longer the impacts last. The messages conveyed in health promotion programmes can be repeated regularly during the school period [8]. Besides helping children to develop personal skills to choose a healthy lifestyle, oral health promotion may support the creation of a healthy school environment [8, 47, 48]. It is suggested that school-based oral health programs with multiple levels of influence may advance oral health equity [10].

One of the considerations in designing health education is the age group of the target population. In preschools, OHE sessions that were delivered through fun activities (i.e., via games, drama) were more effective in improving children’s oral hygiene [21], knowledge, and skills [20] than the traditional OHE. Activities designed to match children’s developmental levels and interests allow them to learn faster. Through playing, children’s motor and cognitive processes of learning progress more rapidly and at an advanced level [20]. Moreover, OHE that is given not only for the children but also for the teachers and parents, will encourage children to adopt a good OHB both at school and home. It was found that a comprehensive programme consisting of OHE sessions to children, teachers, and parents, and supervised tooth brushing with fluoride toothpaste, improved children’s OHB and OHS [17–19]. A professional cross-brushing on first permanent molar surfaces was also found to reduce caries [23].

Similarly, among elementary young students, a programme involving OHE for children, teachers, and parents, was the most effective [11, 25, 28]. In terms of educators, a dentist-led, a teacher-led, and a peer-led OHE were equally effective in improving OHK and oral hygiene status, but the peer-led OHE was better than the teacher-led OHE in enhancing OHB [27]. Another study, however, gave more emphasis to the importance of repetition and reinforcement in OHE than to the educators [29]. The effectiveness of combined approaches of OHE and other interventions, such as the provision of preventive and restorative care, fluoride toothpaste, fluoridated drinking water, a tour of a dental hospital, and competition were also observed in several studies [12, 37, 38]. School dental screening, followed by a series of communication to encourage parents into taking their children to the dentists was effective in improving dental attendance [34].

The positive impacts of tooth-brushing activities were well-demonstrated [30, 31, 33], except for a study in Myanmar that found no impacts following the programme. It was suggested that the factors behind these findings might be the teachers’ lack of skills in giving the instructions as they were not dental professionals, the fact that instructing some groups of young children were not that effective, and children under ten years’ lack of ability to brush [32]. Another type of intervention was a SOC-based intervention, which was found to improve OHRQoL, SOC [35, 36], gingival health, and oral health beliefs [36]. SOC might influence health through physiological (less stress, less physical or biological effects), behavioural (selection of favorable behaviours), and emotional (better ability to cope with stress) pathways [36]. The effectiveness of this intervention was consistently reported in two studies from different countries (i.e., Brazil and Thailand) [35, 36].

Among adolescents, the educational poster was effective in improving knowledge. Nonetheless, the follow-up period in this study was only two weeks [39]. In terms of message framing, loss framing was better than gain framing in encouraging OHB among Iranians. It is worth mentioning, however, that the effects of message framing may depend on the cultural backgrounds, varying between countries [40]. The importance of repetition and reinforcement in OHE, as well as the provision of oral hygiene aids, were also demonstrated [41, 43]. Close monitoring was especially needed when unfamiliar oral hygiene procedures were introduced [42]. An intervention that is noted to be more effective than the traditional OHE for adolescents was motivational interviewing, which was a person-centered counseling strategy [44]. Meanwhile, a programme involving dental hygienists in Sweden was found to have limited impacts on caries incidence, knowledge, and attitudes, but improved adolescents’ interest in oral health. It was suggested that the participants had already had a favourable knowledge and attitude, and a low caries prevalence at baseline, making further improvement difficult to achieve [45].

In summary, most studies found that the intervention programmes brought positive outcomes, especially those involving OHE for children, teachers, and parents, supervised toothbrushing, and provision of fluoride toothpaste and toothbrush. The role of repetition and reinforcement in OHE is highlighted, which is possible through continuous programmes. It may also be beneficial to deliver OHE to pre-schoolers through fun activities. Besides the teacher, parental involvement plays a role in determining the success of the programmes, which may indicate the need to conduct oral health training for them. Future studies that assess the efficacy of home-based oral health promotion programs among children and adolescents will be useful to provide more evidence in developing integrated oral health promotion programmes.

## Supporting information

S1 Checklist PRISMA 2009 checklist. (DOC)
